# Secreted HMGB1 from Wnt activated intestinal cells is required to maintain a crypt progenitor phenotype

**DOI:** 10.18632/oncotarget.10076

**Published:** 2016-06-15

**Authors:** Karen R. Reed, Fei Song, Maddy A. Young, Nurudeen Hassan, Daniel J. Antoine, Nesibe-Princess B. Gemici, Alan R. Clarke, John R. Jenkins

**Affiliations:** ^1^ European Cancer Stem Cell Research Institute, School of Biosciences, Cardiff University, Cardiff, CF24 4HQ, UK; ^2^ Infrafrontier GmbH, Neuherberg / München, 85764, Germany; ^3^ Institute of Translational Medicine, University of Liverpool, Liverpool, L69 3BX, UK; ^4^ Cardiff School of Health Sciences at Cardiff Metropolitan University, Cardiff, CF5 2YB, UK

**Keywords:** Hmgb1, colorectal cancer, intestinal stem cells, Apc, Wnt signalling

## Abstract

**Background and Aims:**

Colorectal cancer (CRC) arises via multiple genetic changes. Mutation of the tumour suppressor gene *APC*, a key regulator of Wnt signalling, is recognised as a frequent early driving mutation in CRC. We have previously shown that conditional loss of *Apc* within the murine small intestine (*Apc^flox^*mice) results in acute Wnt signalling activation, altered crypt-villus architecture and many hallmarks of neoplasia. Our transctipomic profiling (Affymetrix Microarrays) and proteomic profiling (iTRAQ-QSTAR) of Apc-deficient intestine inferred the involvement of High Mobility Group Box 1 (*Hmgb1*) in CRC pathogenesis. Here we assess the contribution of HMGB1 to the crypt progenitor phenotype seen following *Apc* loss.

**Results:**

Elevated HMGB1 was confirmed in intestinal epithelia and serum following conditional loss of *Apc*. Treatment of *Apc^flox^* mice with anti-HMGB1 neutralising antibody significantly reduced many of the crypt progenitor phenotypes associated with *Apc* loss; proliferation and apoptosis levels were reduced, cell differentiation was restored and the expansion of stem cell marker expression was eradicated.

**Methods:**

Hmgb1 levels in intestinal epithelia and serum in *Apc^flox^* and *Apc^Min^* mice were assessed using qRT-PCR, Western blot and ELISA assays. The functional importance of elevated extracellular Hmgb1 was assessed using an anti-HMGB1 neutralising antibody in *Apc^flox^* mice.

**Conclusions:**

HMGB1 is expressed and secreted from intestinal epithelial cells in response to Wnt signalling activation. This secreted HMGB1 is required to maintain nearly all aspects of the crypt progenitor phenotype observed following *Apc* loss and add to the body of accumulating evidence indicating that targeting HMGB1 may be a viable novel therapeutic approach.

## INTRODUCTION

Wnt signalling is a key pathway which regulates normal intestinal homeostasis [1]. It is widely recognised that deregulation of the Wnt pathway underpins the early stages of colorectal cancer [2, 3]. Genetically modified mouse models in which the key Wnt regulator and tumour suppressor gene *Adenomatous polyposis coli* (*Apc*) has been rendered inactive, have greatly assisted in identifying factors that contribute to the pathogenesis of CRC (reviewed [4]). We have previously characterised the murine intestine following the conditional deletion of *Apc*, and shown a rapid perturbation of differentiation, migration, proliferation, and apoptosis accompanying the acute activation of Wnt signalling, such that the Apc-deficient cells maintain a “crypt progenitor-like” phenotype and many hallmarks of neoplasia [5]. Furthermore, we have previously conducted Transcriptomic Affymetrix microarray analysis [5, 6] and proteomic profiling using iTRAQ-QSTAR [7] to identify genes that are mis-regulated at these early stages of neoplasia following the loss of *Apc*, and thereby identify factors that potentially contribute to CRC pathogenesis. These analyses have inferred the involvement of the High Mobility Group Box 1 (*Hmgb1*) gene.

HMGB1 is an abundant multifunctional protein possessing diverse biological activities in normal cells, and has been the subject of intense investigation in recent cancer research being implicated in tumour formation, progression, metastasis and response to chemotherapeutics (reviewed [8]). Within the nucleus, HMGB1 functions to regulate transcription, replication, DNA repair and genomic stability [9–12], while cytoplasmic or cell-membrane bound HMGB1 can regulate cell motility [13–15]. Furthermore, HMGB1 can be released by cells, both passively and actively, whereupon it can act as a cytokine and damage associated molecular pattern (DAMP) molecule [16].

HMGB1 is passively released from cells that have died in a traumatic, non-programmed way (necrosis or pyroptosis) [17, 18]. Significantly, apoptotic cells modify their chromatin so as to bind HMGB1, preventing its release during this form of programmed cell death [17]. Several cell types (including activated immune cells; monocytes, macrophages and natural killer cells) have the ability to actively secrete HMGB1, via a dedicated pathway, and thus produce a damage signal without associated cell death [19]. Once released, extracellular HMGB1 can interact with a number of different receptors including TLR2, TLR4 and RAGE to elicit different responses (reviewed [16, 20]). Post-translational modifications of HMGB1, particularly in relation to the redox state of three cysteines, can influence this receptor binding [20]. The all-thiol form of HMGB1 (in which the cysteines are reduced) is the predominant intra-cellular form and it is this form that is passively released following tissue damage. Once released, all-thiol HMGB1 form elicits the chemo-attractant activities of HMGB1, via the CXCR4 receptor, but this form is not capable of inducing cytokine production [21]. Conversely, disulphide-HMGB1 promotes activation of the NF-κB pathway and proinflammatory cytokine production in fibroblasts and macrophages [21]. Both the all-thiol and disulphide forms of HMGB1 are actively secreted by immune cells, but oxidation of the extra-cellular all-thiol form by reactive oxygen species (ROS) results in accumulation of disulphide and oxidised forms of HMGB1 [21].

Elevated levels of HMGB1 have been reported for numerous cancers, including CRC, and higher levels of HMGB1 has been show to positively correlate with tumour invasion and metastasis (both distant and lymph-node), and reduced survival for CRC patients [22, 23]. Indeed, 7 out of 14 datasets in the Oncomine database display a >2 fold up-regulated of HMGB1 (p<0.05) for the cancer v normal comparison for colorectal cancer, while, one of the four cohorts detailed on Prognoscan (contributed by Staub) displays a statistical significant association between the levels of HMGB1 and clinical outcome (high levels of HMGB1 being associated with a poor prognosis. Both databases accessed 25/5/16). Moreover, elevated levels of serum HMGB1 are associated with the onset of inflammation following the administration of dextran sulfate sodium salt (DSS) in a mouse model of colitis-associated cancer (DSS-treated *Apc^Min^*mice) [24]. Administration of a neutralizing anti-HMGB1 antibody in this mouse model markedly reduced the level of intestinal inflammation and decreased tumour incidence and size, thereby highlighting the potential usefulness of HMGB1 as a target for the treatment of colitis and the prevention of colitis-associated cancer [24]. Furthermore, loss of the RAGE receptor significantly impacts on intestinal tumourigenesis in *Apc^Min^* mice [25].

Here we demonstrate that elevated levels of HMGB1 occur within the intestine and the serum, following the conditional deletion of *Apc* and acute activation of Wnt signalling in the mouse intestine (using *Apc^flox^* mice). We further show that treatment of *Apc^flox^* mice with a neutralizing anti-HMGB1 antibody restores many of the aberrant features associated with loss of *Apc* toward normal. The exact mechanisms by which HMGB1 regulates Wnt signalling and intestinal stem cells remains to be determined, however, our work adds to the accumulating evidence implicating HMGB1 has potential for cancer therapy.

## RESULTS

### Wnt signalling activation results in up-regulated *Hmgb1* expression

**Figure 1:**
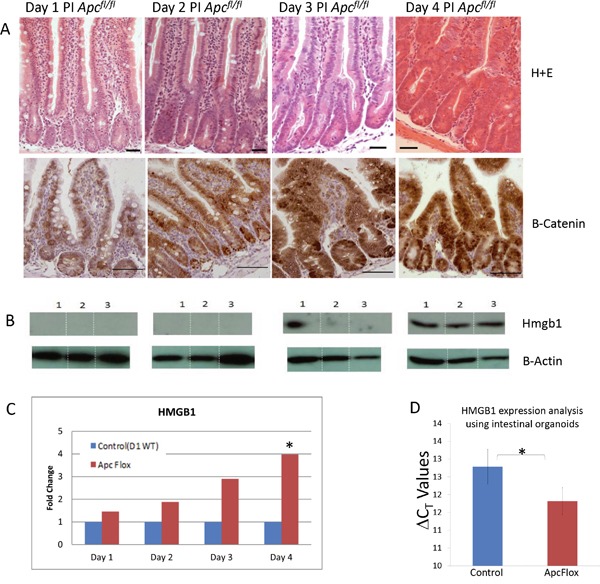
Accumulation of intestinal *Hmgb1* following the loss of *Apc* **A.** H+E stained intestine and β-catenin IHC following the conditional loss of *Apc*. Days post induction are indicated at the top. Elevated nuclear accumulation of β-catenin occurs from day 3 PI onward. **B.** Western Blot analysis of HMGB1 levels on triplicate epithelia cell extracts from each time point. Elevation of HMGB1 was first seen at day 3 PI. **C.** qRT-PCR of *Hmbg1* expression levels presented as relative fold change within epithelia cell extracts compared to day 1 WT demonstrates a significant (*) induction 4 days PI (determined using Whitney U test of ΔCT values at P < 0.05). **D.** qRT-PCR of *Hmbg1* expression levels presented as ΔCT values from control (wildtype) and Apc deficient intestinal organoid cultures. The values shown represent a 1.9 fold over-expression of Hmgb1 in the Apc deficient organoids.

Apc is a known key regulator of Wnt signalling, and critically important in regulating normal intestinal homeostasis. Cre-LoxP driven recombination of *Apc* within the mouse intestine using an Ah-Cre recombinase to drive recombination of LoxP flanked *Apc* alleles, has previously been shown to result in acute activation of Wnt signalling and many hallmarks of neoplasia, including increased proliferation and apoptosis and loss of differentiation and migration [5]. Our previous Affymetrix microarray analysis [5, 6] and proteomic profiling using iTRAQ-QSTAR [7] analysis inferred the involvement of High Mobility Group Box 1 (*Hmgb1*). Subsequently, upregulation of *Hmgb1* expression and elevation of HMGB1 within the intestinal epithelia was confirmed and shown to occur concomitantly with nuclear accumulation of β-catenin (and hence Wnt signalling activation) following the conditional loss of *Apc* (Figure 1). It is of interest to note that a Tcf binding element (TBE sequence [at][at]CAA[at]G) can be found within the human HMGB1 promoter [26], which combined with our data indicates *Hmgb1* is likely a Wnt target gene.

**Figure 2:**
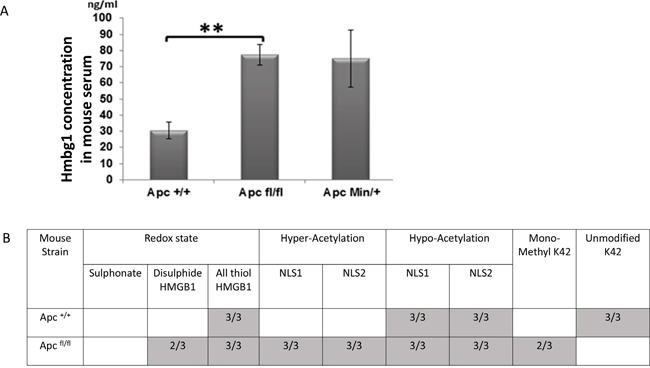
Elevation of serum HMGB1 following the loss of Apc **A.** ELISA analysis demonstrating significant elevation of HMGB1 serum levels in *Apc^flox^* mice and increased levels in *Apc^Min^* mice compared to aged match mice. **B.** Table showing the number of samples in which the different post-translational forms of HMGB1 were detected in the serum of *Apc^flox^* mice day 4 PI, using MS/MS analysis. Empty boxes indicate this form was not detected in any of the samples analysed (n=3).

Different post-translational modifications of HMGB1 are known to be associated with different modes of release, and extra-cellular functions [21, 27]. ELISA analysis confirmed the significant elevation of HMGB1 levels in mouse serum day 5 post induction of *Apc* loss (Figure 2A), although the cellular source for this secreted HMGB1 is not known. These levels are similar to those seen in aged (approximately 6 months old) *Apc^Min^* mice possessing an intestinal tumour burden, but due to a greater variability in aged *Apc^Min^* mice, significant elevation in these samples could not be proven (Figure 2A). To further characterise the form of the secreted HMGB1, tandem mass spectrometry (MS/MS) analysis from *Apc^+/+^* (control) and *Apc^fl/fl^* serum, Day 4 post induction was undertaken (Figure 2B). This demonstrated the acquisition of hyper-acetylated and methylated forms of HMGB1 following *Apc* loss, both of which are forms that are associated with release due to inflammasome activation, pyroptosis and leukocyte and neutrophile derived HMGB1 (reviewed [27]). Thus, activation of Wnt signalling within the intestinal epithelia leads to the active secretion of HMGB1, and significantly higher levels of HMGB1 in the serum.

### Neutralising anti-HMGB1 treatment mitigates the *Apc^flox^* phenotype and reduces pro-inflammatory signals

**Figure 3:**
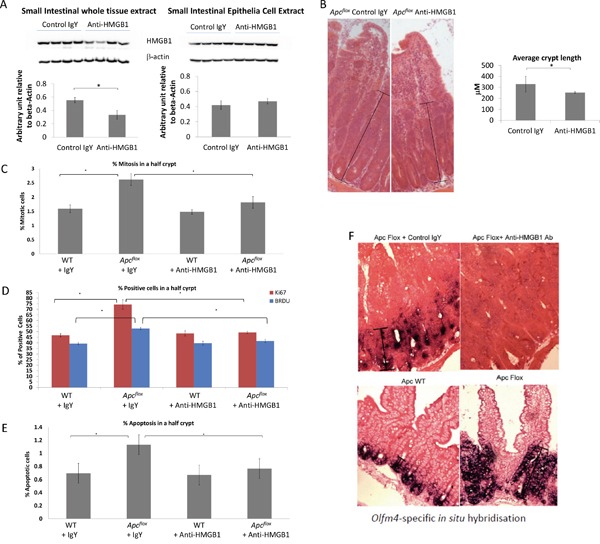
Characterisation of Wnt activated intestine following neutralising HMGB1-antibody treatment **A.** Western blot analysis for total HMGB1 levels in the whole intestine and epithelial cell extracts. Densitometry analysis shows a significant (40%) reduction of HMGB1 protein level in the small intestine tissue, but no change in the epithelia cell extracts small intestine epithelial cells. **B.** The length from the base of a crypt to the top of the aberrant morphology displaying crypt-like features was significantly shorter following the treatment with neutralising HMGB1-antibody. **C.** Percent of mitotic bodies within the “floxed” region, **D.** percent of BRDU and Ki67 positive cells within the “floxed” region, and **E.** percent of apoptotic bodies within the “floxed” region are all significantly reduced following neutralising HMGB1-antibody treatment. **F.** *Olfm4 In situ* Hybridisation shows a marked reduction within the “floxed” region in *Apc^flox^* mice following neutralising HMGB1-antibody treatment. In all cases * denotes p<0.05, M-W U test.

**Figure 4:**
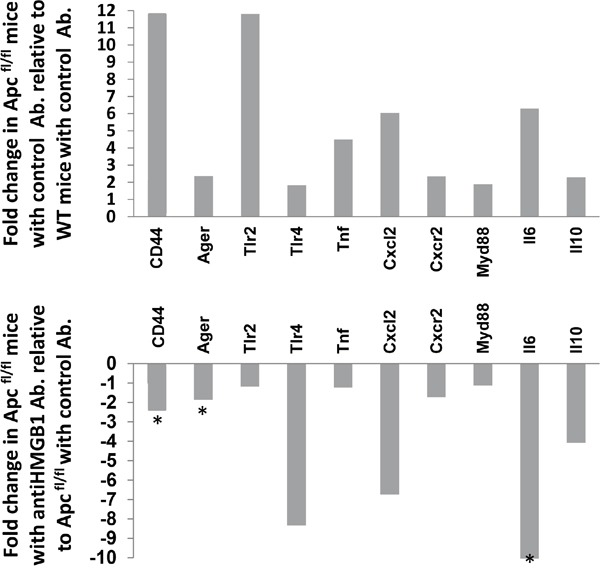
Expression analysis of inflammatory response genes in Wnt activated intestine following neutralising HMGB1-antibody treatment Relative fold-change of a panel of inflammatory response genes in whole intestinal extracts following the loss of *Apc* compared to control (top panel) and in *Apc^flox^* mice following neutralising HMGB1-antibody treatment compared to control IgY treatment. * denotes significance determined using Whitney U test of ΔCT values at P < 0.05.

The functional significance of the elevated extra-cellular HMGB1 levels seen following intestinal Wnt signalling activation was assessed by treating induced *Apc^flox^* mice with neutralising anti-HMGB1 antibody for two days prior to sample collection. Although this treatment regime did not alter the levels of HMGB1 within intestinal epithelial cell extracts, the levels of total HMGB1 seen within the whole intestine was significantly reduced (Figure 3A). Characterisation of the intestinal histology revealed that the extent of the “floxed” region associated with the loss of *Apc* was significantly reduced following neutralising anti-HMGB1 antibody treatment (Figure 3B). Furthermore, microscopic scoring and Immunohistochemical (IHC) analysis clearly showed a significant reduction in the level of epithelia cell proliferation (Figure 3C, 3D) and apoptosis (Figure 3E) within the “floxed” region, while the activation of stem cell markers was significantly reduced (Figure 3F) and goblet cell differentiation was restored to normal (Supplementary Figure 1). Thus, the extra-cellular levels of HMGB1 positively contribute to the phenotype observed following the loss of *Apc* within the intestinal epithelia. qRT-PCR analysis has shown a significant reduction in the HMGB1 receptor RAGE (1.8 fold down), in the whole intestinal tissue from *Apc^flox^* treated with neutralising anti-HMGB1 antibody compared to those treated with control IgY (Figure 4). The importance of the HMGB1-RAGE signalling pathway in initiation of intestinal tumourigenesis has previously been described and a positive feedback loop between ligand and receptor signalling proposed [25]. Our results corroborate these findings, showing reduction in the levels of the ligand HMGB1, results in the reduction in the levels of RAGE receptor, and an overall normalisation of the Wnt signalling induced “crypt-Progenitor like” phenotype. Furthermore, qRT-PCR analysis using whole intestinal tissue has shown a significant reduction in the pro-inflammatory marker and Wnt signalling target gene CD44 (2.7 fold down) and cytokine Il6 (10.5 fold down) in the *Apc^flox^* treated with neutralising anti-HMGB1 antibody compared to those treated with control IgY (Figure 4). However, generic alteration of Wnt signalling target genes was not seen; levels of *cMYC*, *PPARdelta, Tcf7, Lef1, Fgf4, MMP7* and *Wisp1* were not affected by the neutralising anti-HMGB1 antibody in the *Apc^flox^* intestine.

## DISCUSSION

Here we show that activation of Wnt signalling, a common and often early event in intestinal tumourigenesis, results in the up-regulation and active secretion of Hmgb1 within the intestine, which in turn results in elevated levels of serum Hmgb1. It is likely that release of HMGB1 signals via the RAGE receptor and promotes inflammation activation, which in turn feeds-back to the mechanisms regulating normal intestinal homeostasis and the stem cell compartment within the intestinal epithelia. The process of inflammation driven intestinal repair is known to promote a tumour-promoting environment [24, 28], and the release of HMGB1 contributes to tumourigenesis in mouse models of colitis-associated cancer [24, 25, 29].

However, we have also shown that elevated levels of Hmgb1 contribute towards many of the abnormal “crypt progenitor” phenotypes that occur following the loss of *Apc*, including the expansion of intestinal stem cell marker expression. The exact mechanisms by which Hmgb1 influences the intestinal stem cells remains to be elucidated, but the lack of generic alteration of Wnt signalling target genes following the neutralising antibody treatment despite the fact that HMGB1 has also been shown to be a “non-core regulator” and strong inducer of Wnt/TCF-dependent transcription [30], suggests altered Wnt signaling is not the likely underlying mechanism. Rather the influence of Hmgb1 on immune cells and the inflammatory response could be a contributing factor. However, our work provides evidence that Hmgb1 is a potential Wnt signalling target gene and contributes to the body of evidence implicating Hmgb1 in cancer formation with the potential for exploitation as a therapeutic target.

## MATERIALS AND METHODS

### Mice and sample preparation

This study received ethical approval from Cardiff University's Animal Welfare and Ethical Review Body (previously known as the ERP), and all animal procedures were conducted in accordance with UK Home Office regulations. *AhCre^+^*Apc*^+/+^* (control) and *AhCre^+^*Apc*^fl/fl^* mice were generated and maintained on an outbred background as previously described [5]. Cre-recombinase activity was induced from the Ah-Cre transgene by three intra-peritoneal (IP) injections of 80 mg/kg β-naphthoflavone within 24 h on day 0. Mice were sacrificed, and samples collected on the appropriate day post induction as previously described [5].

Mice received 225 mg of either anti-Hmgb1 antibody or the control IgY (products ST326052233, IBL International GmbH), IP daily on days 2 and 3 post-induction, and sacrificed on day 4 (n=4 in each cohort). Where appropriate, 250μl BrdU (Amersham) was administered via IP injection 2 hrs before sacrifice.

### Phenotypic characterisation of intestinal sections

Immunohistochemistry (IHC) was performed on paraffin embedded tissues fixed in 4% formaldehyde at 4°C for no more than 24 hours prior to processing. The following antibodies were used for IHC: BrdU 1:500 BD biosciences; Ki671:100 Vector Labs ; Caspase 3 1:750 R&D systems; β-catenin 1:50 C19220, Transduction Laboratories.

Crypt length from the base of a crypt to the top of the aberrant morphology displaying crypt-like features was measured along 50 clear crypt/villus axes for each sample. Apoptosis and mitotic index were scored from H&E stained sections as previously described [5].

### Western analysis

Protein was extracted (from 50-100 mg tissue) using RIPA lysis buffer and protein concentrations determined (BCA kit, Pierce). 60μg of cellular protein was separated on a 10% polyacrylamide gel and transferred to nitrocellulose (Schleicher&Schuell). Following a blockinf step (TBS containing 5% non fat dry milk, 2% donkey serum, 0.02% TX-100, 0.02% NaN3) the following antibodies were used: anti-HMGB1 1:1,000 (TECAN), mouse anti-actin (ICN) 1:1,000; HRP-conjugated secondary donkey antibodies (Diagnostics Scotland) 1:5,000. SuperSignal West Femto ECL reagent (Pierce) was used for detection.

### qRT_PCR

RNA extracted from whole intestinal samples, was used to synthesise first strand cDNA using a VersoTM cDNA Kit (Thermo Scientific) and anchored oligo-dT primers (Thermo Scientific) according to the manufacturer's instructions. All qRT-PCR reactions were run on the Applied Biosystems StepOnePlus machine. The programming was carried out using the computer-based StepOnePlus software. Primers were designed using the Universal ProbeLibrary Assay Design Centre (Roche).

**Table:** 

Gene Name	FWD sequence	REV sequence
beta-Actin	ctaaggccaaccgtgaaaag	accagaggcatacagggaca
CD44	tccttctttatccggagcac	acgtctcctgctgggtagc
Ager	ggtccactggataaaggatgg	taggtgccctcatcctcgt
Tlr2	ggggcttcacttctctgctt	agcatcctctgagatttgacg
Tlr4	ggactctgatcatggcactg	ctgatccatgcattggtaggt
Tnf	tcttctcattcctgcttgtgg	ggtctgggccatagaactga
Cxcl2	aaaatcatccaaaagatactgaacaa	ctttggttcttccgttgagg
Cxcr2	caggaccaggaatgggagta	tcccctccaaatatccccta
Myd88	tgacttccagaccaagtttgc	gaatcagtcgcttctgttgga
Il6	tctaattcatatcttcaaccaagagg	tggtccttagccactccttc
Il10	cagagccacatgctcctaga	gtccagctggtcctttgttt
Hmgb1	ttgggtcacatggattattagtgt	cagggcatgtggacaaaag

### ELISA and MS/MS analysis

Immediately following cervical dislocation, blood was collected directly from the chest cavity and transferred into anti-clotting tubes (Sarstedt), kept on ice for a minimal time, then centrifuged at full speed for 2min to separate serum and cell pellets. Serum was transferred into fresh tubes and snap-frozen in liquid nitrogen. HMGB1 protein in serum was quantified by ELISA (Shino-Test Corp.) following the manufacturer's instructions. Mass Spectrometric characterisation of HMGB1 (MS/MS) was conducted as previously described [31–33].

## SUPPLEMENTARY FIGURE



## List of abbreviations used (if any)

CRC: Colorectal cancer
*APC*: *Adenomatous polyposis coli*
*Hmgb1*: High Mobility Group Box 1
MS/MS: Tandom Mass spectrometry
TBE: *Tcf binding element*
IP: Intra-peritoneal
IHC: Immunohistochemistry
DSS: dextran sulfate sodium salt
ROS: reactive oxygen species
