# Supplementary material for: Secreted HMGB1 from Wnt activated intestinal cells is required to maintain a crypt progenitor phenotype
Source: Oncotarget. 2016 Jun 15;7(32):51665–73. doi: 10.18632/oncotarget.10076 (PMC5239505; doi:10.18632/oncotarget.10076)
Supplement: Supplementary file 1 [file oncotarget-07-51665-s001.pdf]

Secreted HMGB1 from Wnt activated intestinal cells is required to maintain a crypt progenitor phenotype

SUPPLEMENTARY FIGURE

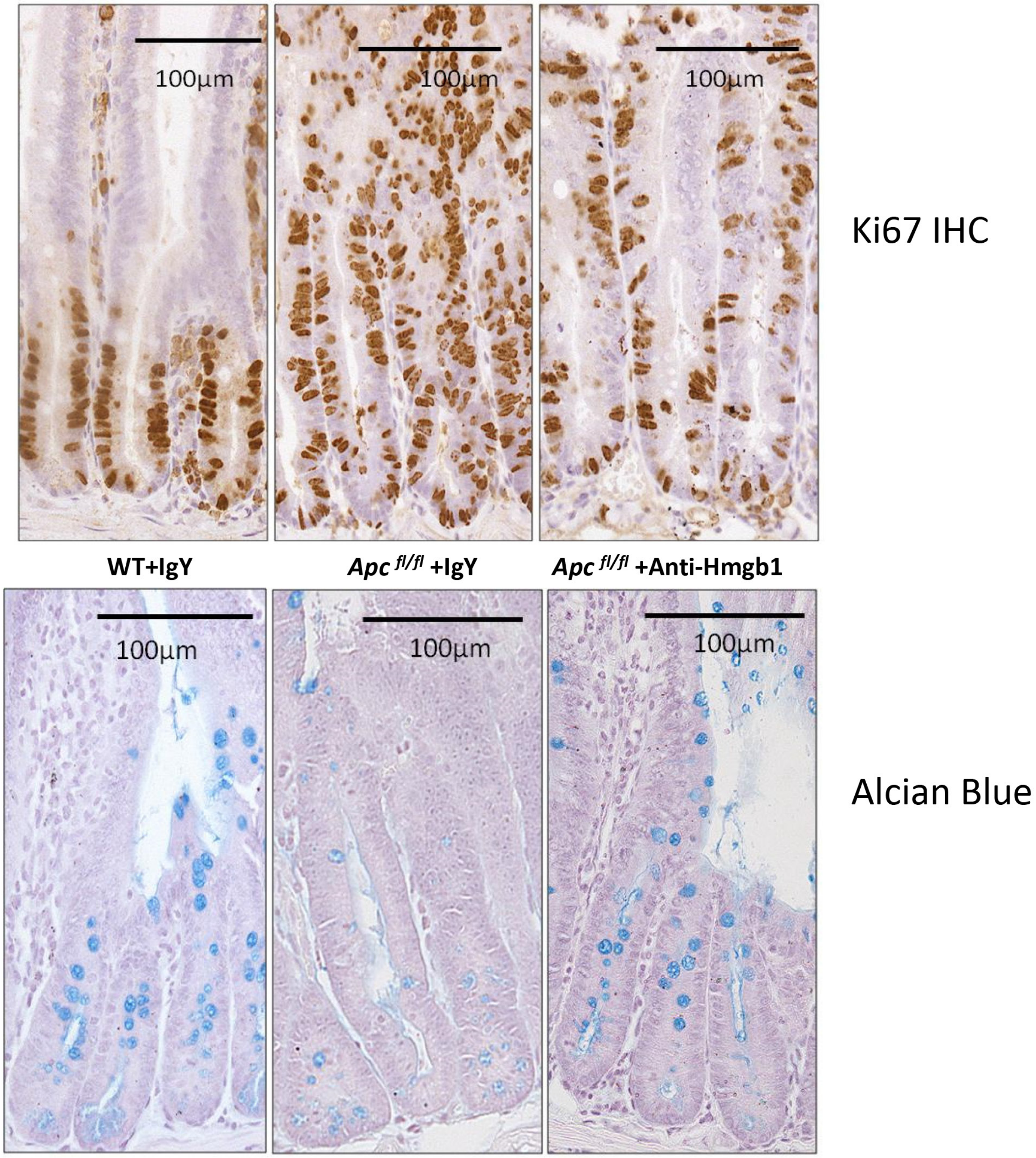

Supplementary Figure 1: Cell proliferation and differentiation following neutralising HMGB1-antibody treatment. IHC analysis for Ki67 and Alcian blue staining for Goblet cell differentiation on intestinal sections of control (WT) and induced ApcFlox day 4 post induction treated with IgY or anti-HMGB1 antibodies. Note the comparative reduction in levels of Ki67 and increased numbers of goblet cells in the anti-HMGB1 treated ApcFlox sections.
